# Novel algorithms for improved detection and analysis of fluorescent signal fluctuations

**DOI:** 10.1007/s00424-023-02855-3

**Published:** 2023-09-13

**Authors:** Gebhard Stopper, Laura C. Caudal, Phillip Rieder, Davide Gobbo, Laura Stopper, Lisa Felix, Katharina Everaerts, Xianshu Bai, Christine R. Rose, Anja Scheller, Frank Kirchhoff

**Affiliations:** 1 Department of Molecular Physiology, Center for Integrative Physiology and Molecular Medicine (CIPMM), University of Saarland, Building 48, 66421 Homburg, Germany; 2 Institute of Neurobiology, Faculty of Mathematics and Natural Sciences, Heinrich Heine University Düsseldorf, 40225 Düsseldorf, Germany

**Keywords:** Calcium signal analysis, ROI detection, Background correction, Transient classification, Interactive user interface, Glial calcium signals, Neuronal SBFI imaging

## Abstract

Fluorescent dyes and genetically encoded fluorescence indicators (GEFI) are common tools for visualizing concentration changes of specific ions and messenger molecules during intra- as well as intercellular communication. Using advanced imaging technologies, fluorescence indicators are a prerequisite for the analysis of physiological molecular signaling. Automated detection and analysis of fluorescence signals require to overcome several challenges, including correct estimation of fluorescence fluctuations at basal concentrations of messenger molecules, detection, and extraction of events themselves as well as proper segmentation of neighboring events. Moreover, event detection algorithms need to be sensitive enough to accurately capture localized and low amplitude events exhibiting a limited spatial extent. Here, we present two algorithms (PBasE and CoRoDe) for accurate baseline estimation and automated detection and segmentation of fluorescence fluctuations.

**Supplementary Information:**

The online version contains supplementary material available at 10.1007/s00424-023-02855-3.

## Introduction

Analysis of ligand-dependent fluorescence fluctuations in the central nervous system (CNS) is a major step in unravelling complex regulatory functions. Astroglial Ca^2+^ events are considered a key factor in identifying the versatile roles of astrocytes in health and disease [2, 3, 8, 13]. However, reliable detection, analysis, and interpretation of such Ca^2+^ events are non-trivial tasks [1, 9, 11, 14, 27, 31, 33]. One of the challenges in analyzing astroglial Ca^2+^ events originates from the heterogeneous nature of astrocytes themselves, reflected in a complex morphology, diverse interactions with other cell types ranging from vasculature to synapses, and the formation of intercellular gap-junction coupled syncytia [8, 24, 25]. The majority of astroglial Ca^2+^ events occur in fine and highly ramified astroglial processes [6], localized at end feet [29] or at synaptic compartments [1]. These so-called microdomain events are elementary signals, serving autonomous functions [30]; they are not location specific and can occur at all regions of the complex process network. Occurring in functionally independent cellular sub compartments, they occupy volumes in the sub-μm^3^ range and cause changes in fluorescence close-to-noise level [6, 23], rendering these events particularly difficult to characterize, requiring careful detection and removal of fluorescence background [23].

Fluorescence events are classically analyzed using stationary regions of interest (ROIs). Recently, new approaches utilizing so-called dynamic events [7, 23, 33] to extract and analyze non-stationary fluorescence events, capturing morphological changes of the fluorescent event itself, have been proposed. This poses an important extension to the classic ROI-based analysis, capable of revealing new insights. However, most fluorescence events, including the majority of astroglial Ca^2+^ events, are stationary [33], exhibiting only small to no changes in event morphology and location. Hence, this work focuses on the continued relevance and effectiveness of classic ROI-based analysis as a robust tool for quantifying fluorescence events. Two novel automated algorithms, called PBasE (**P**olynomial **Bas**eline **E**stimation) and CoRoDe (**Co**rrelation-based **RO**I **De**tection), are presented. Both algorithms are designed to work independent of specific fluorophores and cell types. The aim of PBasE is to accurately estimate basal fluorescence levels (*F*_0_) while simultaneously preserving fluorescence transients originating from phasic signaling. Building upon the background-corrected dataset ( (*F* − *F*_0_)/*F*_0_ = ∆*F*/*F*_0_ ), CoRoDe is aimed at extracting and segmenting fluorescently active regions by explicitly leveraging temporal information. These algorithms are made accessible via a graphical and interactive analysis application, developed in MATLAB [21], called MSparkles (Suppl. Figure 1A). MSparkles itself is developed to aid the analysis process by providing direct visual feedback to the user, allowing to explore datasets, quickly optimizing analysis parameters, and automating cumbersome tasks, such as signal analysis as well as result export.

The capabilities of PBasE and CoRoDe as well as MSparkles are demonstrated by analyzing differences in astroglial Ca^2+^ events, recorded in the somatosensory cortex of awake and anesthetized mice. Such signals have been studied before [7, 23, 32], serving as reference to our results. Next, the results obtained by MSparkles are compared to those obtained with three other Ca^2+^ analysis tools, CHIPS [5], CaSCaDe [1], and AQuA [33]. CHIPS provides a collection of various classes and algorithms for specific events, usable with little programming skills. CaSCaDe combines an activity-based ROI detection with a machine learning approach for event extraction. AQuA is based on machine learning principles and uses a combination of thresholding and probabilistic modeling to extract dynamic events. Finally, the use of MSparkles in analyzing fluorescence events is tested in two different experimental situations—Ca^2+^ signals obtained in GCaMP3 reporter mice, as well as neuronal Na^+^ signals visualized using SBFI-AM.

## Materials and methods

### Cranial window surgery for in vivo two-photon imaging

During surgical procedures, animals were kept on heating pads and eyes were covered with Bepanthen ointment (Bayer). Anesthesia was induced with a mixture of 5% isoflurane, 47.5% O_2_ (0.6 l/ min), and 47.5% N_2_O (0.4 l/ min) and maintained with 2% isoflurane (Harvard Apparatus anesthetic vaporizer). A standard craniotomy [10] of 3 mm in diameter was performed over the somatosensory cortex (2 mm posterior and 1.5 mm lateral to bregma). The craniotomy was sealed with a glass coverslip and fixed with dental cement (RelyX®, 3M ESPE). Subsequently, a custom-made metal holder for head restraining (5 mm diameter) was applied and fixed to the skull with dental cement. After surgery, the animals were kept on the heating pad until complete recovery and received pain treatment for at least 3 days (carprofen/buprenorphine). After 5 to 7 days, the first imaging session was performed.

### Two-photon laser scanning microscopy (2P-LSM)

In vivo 2P-LSM was performed using a custom-built microscope equipped with a resonant scanner (RESSCAN-MOM, Sutter instrument) and a 20× water-immersion objective (W Plan-Apochromat 20x/1.0 DIC D=0.17 M27; Zeiss). Images were acquired with a frame rate of 30 Hz and a frame-averaging factor of 10, resulting in an effective acquisition rate of 3 Hz. Recorded fields of view (FOVs) had a size of 256 × 256 μm, sampled with 512 × 512 pixels (0.5 μm/pixel). To minimize photo-damage, incident laser power was kept between 30 and 40 mW for a sufficient signal-to-noise ratio. Laser wavelength was set to 890 nm (Chameleon Ultra II, Ti:Sapphire Laser, Coherent). The emitted light was detected by photomultiplier tubes (R6357, Hamamatsu) [10] and pre-amplified (DHPCA-100, Femto). Digitizer (NI-5734) and control hardware (NI-6341) were housed in a NI PXIe (1082) chassis, connected to a control PC via a high bandwidth PXIe-PCIe8398 interface. Scanning and image acquisition were controlled by ScanImage (SI 5.6R1) [28].

### In vivo two-photon Ca^2+^ imaging

In preparation for Ca^2+^ imaging, animals were habituated before the first imaging session according to adapted protocols without water restriction [15, 19]. The animals were head-fixed using a custom-designed, 3D-printed stainless-steel head restrainer. During imaging, anesthesia was administered using a custom-made, magnetically attachable mask. Each FOV was imaged twice: first in anesthetized, then in awake state. During imaging in anesthetized state, isoflurane concentration was kept at 1.5%, and flow of O_2_ and N_2_O was set to 0.6 l/min and 0.4l /min, respectively. Before awake imaging, isoflurane and other gases were switched off and it was visually verified that animals were fully awake (mice were grooming, voluntarily moving, or reacting to air puffs). FOVs for Ca^2+^ imaging were located in the somatosensory cortex, 80–100 μm beneath the dura. Each FOV was recorded for 5 min per condition to investigate Ca^2+^ events. The total duration of one imaging session ranged between 30 and 60 min per animal. Anesthetized mice were kept on a heating pad at 37°C until complete recovery. In addition, mice had access to high-caloric food (Fresubin, Fresenius Kabi GmbH) ad libitum.

### Wide field in situ Na^+^ imaging

Balb/c mice aged between postnatal day (P) 14–18 were anesthetized with CO_2_ before being quickly decapitated and 250 μm hippocampal slices were prepared [12, 18]. Slices were then transferred into an experimental bath, continuously perfused with standard, CO_2_/HCO_3_^−^-buffered artificial cerebrospinal fluid (ACSF) and their CA1 region bolus-stained with the Na^+^ sensitive dye SBFI-AM (sodium-binding benzofuran isophthalate–acetoxymethyl ester; Invitrogen, Karlsruhe, Germany) [12, 18]. SBFI was alternatively excited at 340/380 nm at an imaging frequency of 0.5 Hz and emission was collected >440 nm from defined regions of interest (ROIs) reflecting cell bodies of CA1 pyramidal neurons. Changes in the SBFI ratio were transferred into changes in intracellular Na^+^ concentration based on in situ calibrations [12, 18]. Recurrent network activity was induced via perfusion with an ACSF lacking Mg^2+^ and containing 10 μM bicuculline, in order to remove the Mg^2+^ block from NMDA receptors, and to prevent GABA_A_ receptor activation respectively [18].

### Animals

For Ca^2+^ imaging experiments conducted in Homburg, Germany, mice were kept and bred in strict accordance with the recommendations to European and German guidelines for the welfare of experimental animals. Animal experiments were approved by the Saarland state’s “Landesamt für Gesundheit und Verbraucherschutz” in Saarbrücken/Germany (veterinary licenses: 71/2013, 36/2016). Astrocyte-specific knockin GLAST-CreERT2 mice (Slc1a3tm1(cre/ERT2)Mgoe, MGI:3830051) [22] were crossbred to Rosa26 reporter mice with GCaMP3 expression (Gt(ROSA)26Sortm1(CAG-GCaMP3)Dbe, MGI: 5659933 [26]. Imaging sessions were performed at 8–10 weeks of age, 21 days after tamoxifen induced recombination [17]. GCaMP3 expression was induced in Glast-CreERT2 mice; tamoxifen (Carbolution, Neunkirchen, Germany) was intraperitoneally injected (100 μL/10 g body weight with 10 μg/mL tamoxifen in Mygliol®812 (Caesar and Lorentz GmbH, Hilden, Germany)) to mice once per day for three consecutive days.

Na^+^ imaging experiments conducted at the Heinrich Heine University Düsseldorf, Germany, were carried out in accordance with the institutional guidelines and the European Community Council Directive (86/609/EEC). All experiments were approved by the Animal Welfare Office at the Animal Care and Use Facility of the Heinrich Heine University Düsseldorf (institutional act number: O52/05.

### Statistical analysis and figures

Statistical analysis of computed data was conducted using GraphPad Prism 8. D’Agostino-Pearson normality test was used to determine whether values for statistical evaluation were normally or log-normally distributed. For non-normal distributions, median values, with their associated ranges, inter quartile ranges, and percentiles, were used for statistical evaluation. Similarly, statistical significances were computed using the Mann-Whitney test for individual, or the Kruskal-Wallis test for multiple comparisons, as non-parametric tests, suitable for non-normal distributions.

Figures were arranged using Adobe InDesign 2020, Adobe Illustrator 2020, and GraphPad Prism 8. Graphs, trace plots, kymographs, and ROI maps were directly exported from MSparkles. Additional ROI maps were extracted from the respective Ca^2+^ analysis tools.

### Pre-processing

Pre-processing was adjusted individually per dataset as needed using MSparkles built-in pre-processing pipeline (Suppl. figure 1B). Independent of individual settings, all datasets were denoised (SURE-LET [20]) and subjected to a temporal median filter. No external software was used. The pre-processed dataset is referred to as *F*.

## Results

We developed two novel algorithms to improve the automated detection of fluorescence events and their subsequent analysis. For that purpose, we recorded changes of intracellular Ca^2+^ and Na^+^ activities using two-photon laser scanning microscopy. Ca^2+^ events were visualized by transgenic expression of GCaMP3 in astrocytes, while Na^+^ changes were visualized using SBFI-AM in neurons. In addition, we compared the results obtained by the two novel algorithms to results obtained using other analysis applications, analyzing the same datasets. These datasets exhibited a broad range of fluorescence events, ranging from somatic events to events in the gliapil, from dim to bright events, varying spatial extent as well as stationary events, dynamic events as well as global events extending throughout the recorded field of view.

### *F*_0_ estimation

**Fig. 1:**
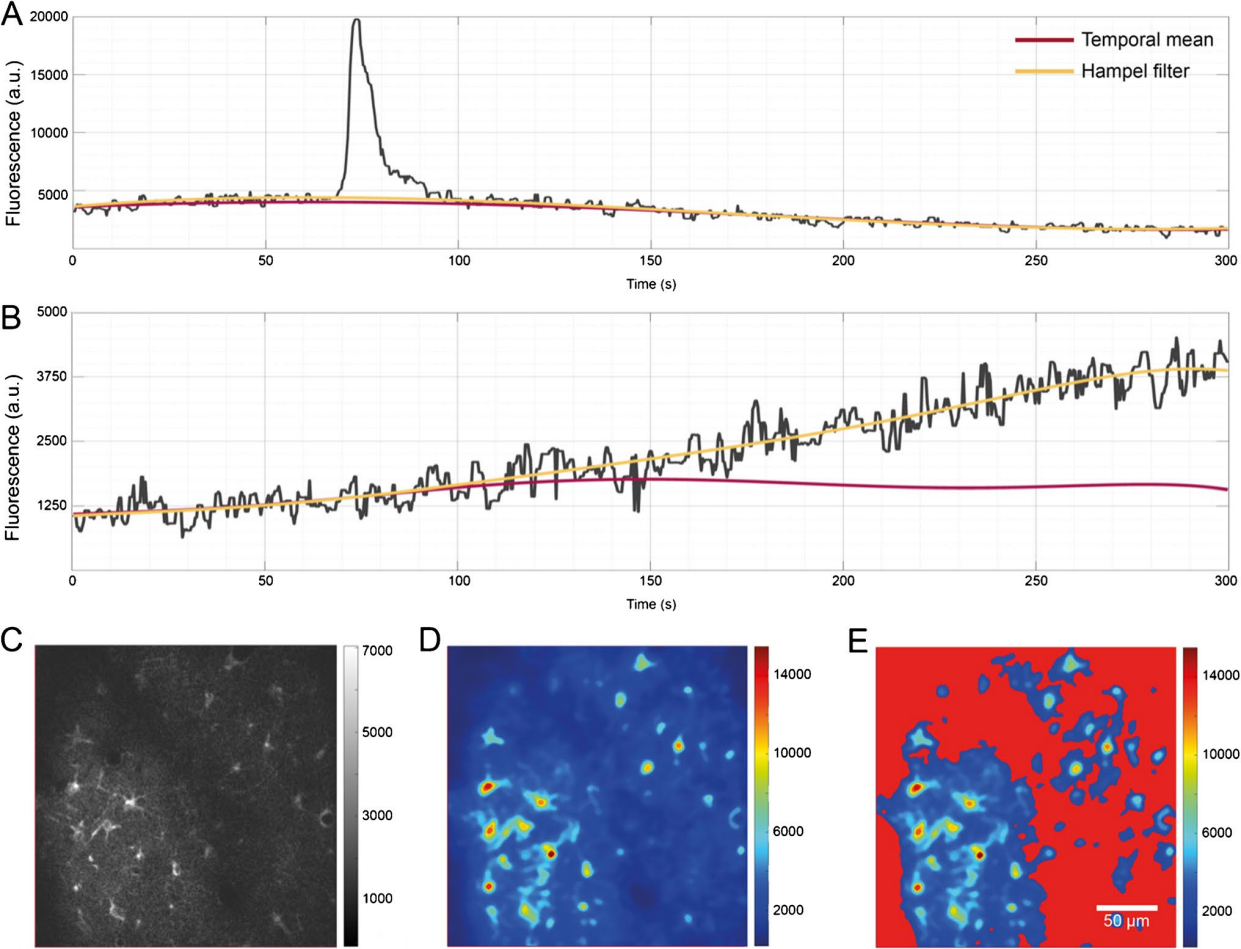
*F*_0_ estimation by polynomial fitting. From the recorded signal of a single pixel (**A**, **B** gray line), transients were excluded for baseline estimation either by a Hampel filter or by using the overall temporal mean of a pixel’s signal. A polynomial is then fitted to the “clean” signal in a least-squares sense to obtain the estimate of the basal fluorescence level (red, *F*_0_ estimation based on temporal mean; yellow, *F*_0_ estimation based on Hampel filter). (**A**) Both methods return similar estimates if no long-lasting and slow increases in basal fluorescence are present. (**B**) The temporal mean filter can preserve long-lasting and slow changes, while the Hampel filter incorporates them into the estimated baseline. (**C**) An individual frame of the original image stack did not reveal fluorescently active regions. (**D**) The fluorescence range projection revealed regions with fluctuations in fluorescence. (**E**) *F*_0_ masking based on the range projection allows to exclude regions with little or no fluorescent activity from the *F*_0_ estimation, and effectively prevented the detection of false-positive ROIs and subsequent false transients

PBasE (**P**olynomial **Bas**eline **E**stimation) is an automated, pixel-based algorithm to estimate fluorescence levels at basal concentrations (*F*_0_) of Ca^2+^ and other important messenger molecules (Fig. 1A and B). It operates solely along the temporal axes of a dataset and is thus equally well suited for datasets containing two as well as three spatial dimensions. First, the algorithm performs signal clean-up and simplification. It thereby excludes statistically large values from the *F*_0_ estimation. This is implemented in two ways. A temporal mean filter computes the mean value (*μ*) and the corresponding standard deviation (*σ*) over a pixel’s entire time course. Then, all values >*μ* + *nσ* are eliminated, where *n* is a user-definable factor. Alternatively, a Hampel filter [16] can be used, which is the sliding window counterpart to the temporal mean filter. To avoid high-frequency oscillations of the fitted polynomial, especially near the beginning and the end of the recorded signal, signal simplification is performed, using piece-wise constant functions to compute a guidance signal. Therefore, a scale space with *k* scales, each containing 2^*k*^ sections, where *k* ∈ [1. . *m*] is computed. Each constant section is computed within an interval $\left[\left(k-1\right)\ast \frac{T}{2^k}+1,\kern0.5em k\ast \frac{T}{2^k}\ \right]$, where *T* is the number of recorded time points. The guidance signal can be optimized with respect to local minima, maxima, or minimal error to the cleaned signal within the intervals. Finally, a polynomial curve of user-definable degree is fitted to the optimized guidance signal in a least-squares sense.

### *F*_0_ masking

Normalizing and detrending a dataset by computing ∆*F*/*F*_0_ may amplify artificially amplified artifacts in dark, noisy image regions with no fluorescence activity. Independent of the method used to estimate *F*_0_, values of 0 < *F*_0_ ≤ 1 can occur. This is likely to amplify noise and may lead to the detection of false-positive events, ultimately resulting in the detection of false transients. This problem is solved by *F*_0_ masking, similar to [4]. For this purpose, the original stack *F* (Fig. 1C) is used to compute the range projection *R* with *R* = max(∆*F*/*F*_0_(*p*)) − min(∆*F*/*F*_0_(*p*)) (Fig. 1D) along the temporal axes. By applying a user-definable threshold to the range projection, requiring a minimal fluorescence change the *F*_0_ mask (Fig. 1E) is obtained. An initial threshold value is estimated using Otsu’s method, instead of using a fixed percentile as in [4]. Pixels covered by the *F*_0_ mask are excluded from the *F*_0_ estimation.

### ROI detection

**Fig. 2:**
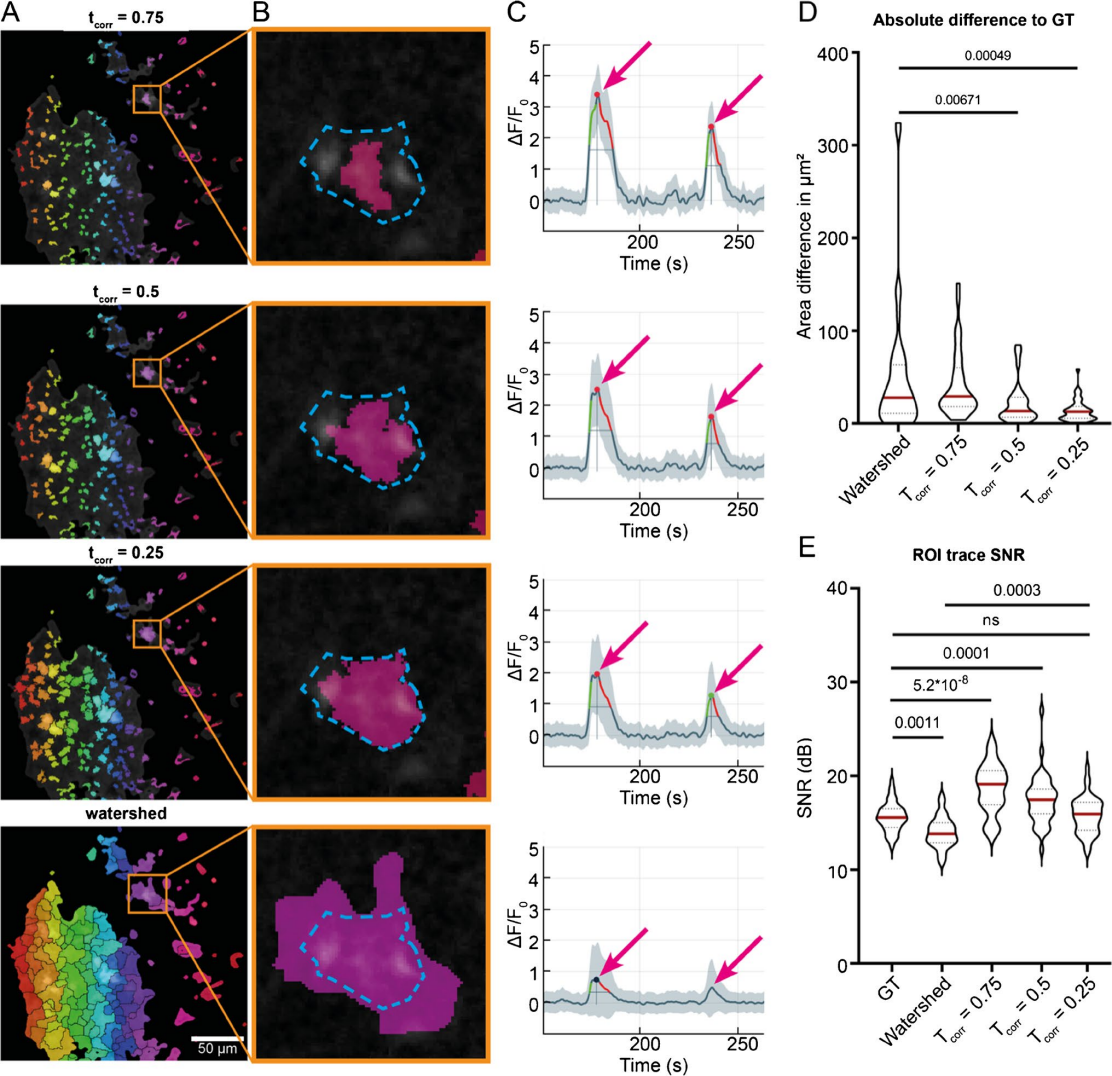
Temporal correlation–based ROI detection. (**A**) ROIs obtained using CoRoDe with different correlation thresholds (*t*_*corr*_) as well as watershed transform. Correlation thresholds were set to 0.75, 0.5, and 0.25. (**B**) Direct comparison of automatically extracted ROIs to a manually evaluated ground truth. The obtained ROIs (pink) were contrasted to the manually extracted maximal extent of the fluorescence event (dashed blue line). (**C**) Fluorescence profiles from the highlighted ROIs showed the influence of accurate segmentation on resulting peak amplitudes. Transients of ROIs obtained by watershed segmentation exhibit too small peak amplitudes, while transient peaks of undersized ROIs were too high (arrows). (**D**) ROIs obtained using CoRoDe showed significantly less difference from ground truth, compared to watershed transform. (**E**) Integrated fluorescence profiles from ROIs with appropriate *t*_*corr*_ show no difference in SNR, compared to ground truth, whereas profiles from ROIs obtained using a watershed transform showed a significantly reduced SNR

Detection and segmentation of events into ROIs are performed by the CoRoDe algorithm (**Co**rrelation-based **RO**I **De**tection, Fig. 2) and are based on the range projection *R*. Pixels excluded by the *F*_0_ mask are zero in *R*. CoRoDe uses simultaneous region growing of local maxima, constrained by a minimally required temporal correlation of neighboring pixels in addition to a range threshold, *t*_*R*_. Local maxima are grown into their neighborhoods until either *t*_*R*_ or the correlation threshold (*t*_*corr*_) is violated. Pixels adjacent to more than one region are marked as boundary pixels. Decreasing *t*_*corr*_ to zero results in region growing being governed by the range threshold, and the detected regions become more similar, but not identical, to those obtained by a watershed segmentation (Fig. 2A, bottom).

### Transient analysis and classification

ROI integration and transient extraction and classification are automatically performed on ∆*F*/*F*_0_ in MSparkles. Also, transient amplitudes, durations, rise, and decay times (Suppl. Figure 2A, Suppl. Table 1) as well as inter-transient timings between consecutive transients are automatically determined (Suppl. figure 2B, Suppl. Table 1), but also ROI area, number of transients, transients per ROI, or overall statistics related to durations, amplitudes, frequencies, or integrated fluorescence values per transient (area under the transient curve). Transient durations can be computed as the full width at half maximum (FWHM), full width at 25% or 10% of the peak amplitude. The latter two can lead to a much more accurate estimation of transient duration but require a higher signal quality. Optionally, sub-transients can be excluded. Sub-transients are non-maximal peaks of a multi-peak transient, i.e., amplitude peaks occurring during the rise or decay of a stronger peak. This strongly depends on the level at which transient duration is computed.

**Fig. 3:**
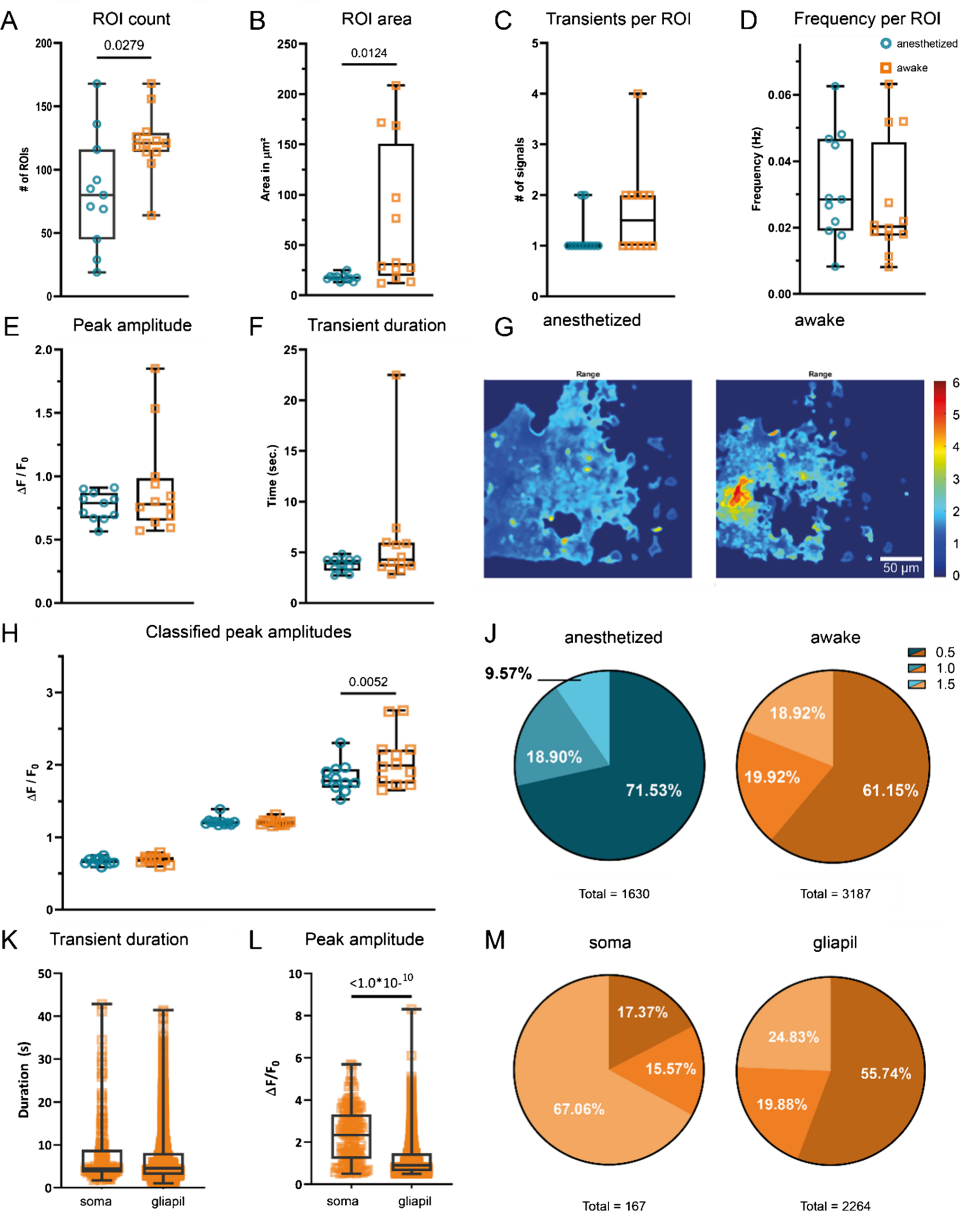
Statistical analysis and transient characterization of Ca^2+^ signals in GCaMP3-expressing mice. The median number of detected ROIs (**A**), as well as the ROI area (**B**), increased in awake mice. The median number of transients per ROI also doubled in awake mice expressing GCaMP3 (**C**). Per-ROI transient frequencies did not change (**D**). The overall median transient peak in awake mice did not change (**E**). Overall median transient duration showed no change in awake mice (**F**). The range projection of ∆*F*/*F*_0_ indicates the amplitude and extent of fluorescence fluctuations (**G**). Individual classes (**H**, **J**) show not only an increase in median amplitude above 1.5 ∆*F*/*F*_0_ (**H**) but also a relative increase (signal composition) in strong transients during wakefulness (**J**). Differential analysis of somatic transients and transients occurring in the gliapil (**K**, **L**, **M**) shows similar median durations (**K**). Somatic transients exhibit not only a higher median peak amplitude (**L**), but also occur mostly as high amplitude transients (**M**), compared to transients in the gliapil

Extracted transients are automatically classified into user-definable classes based on their peak amplitude. By default, three pre-defined classification intervals [0.5..1.0), [1.0..1.5), and [1.5. . ∞) are used. Detected ROIs not exceeding the lowest classification threshold (here 0.5 ∆*F*/*F*_0_) at any time are considered false positives and are automatically removed. The classified transients are used to compute the signal composition, represented by the relative frequency of transients belonging to a specific class (Fig. 3J and M), which reveals changes in signaling behavior.

### Synchronicity analysis

**Fig. 4:**
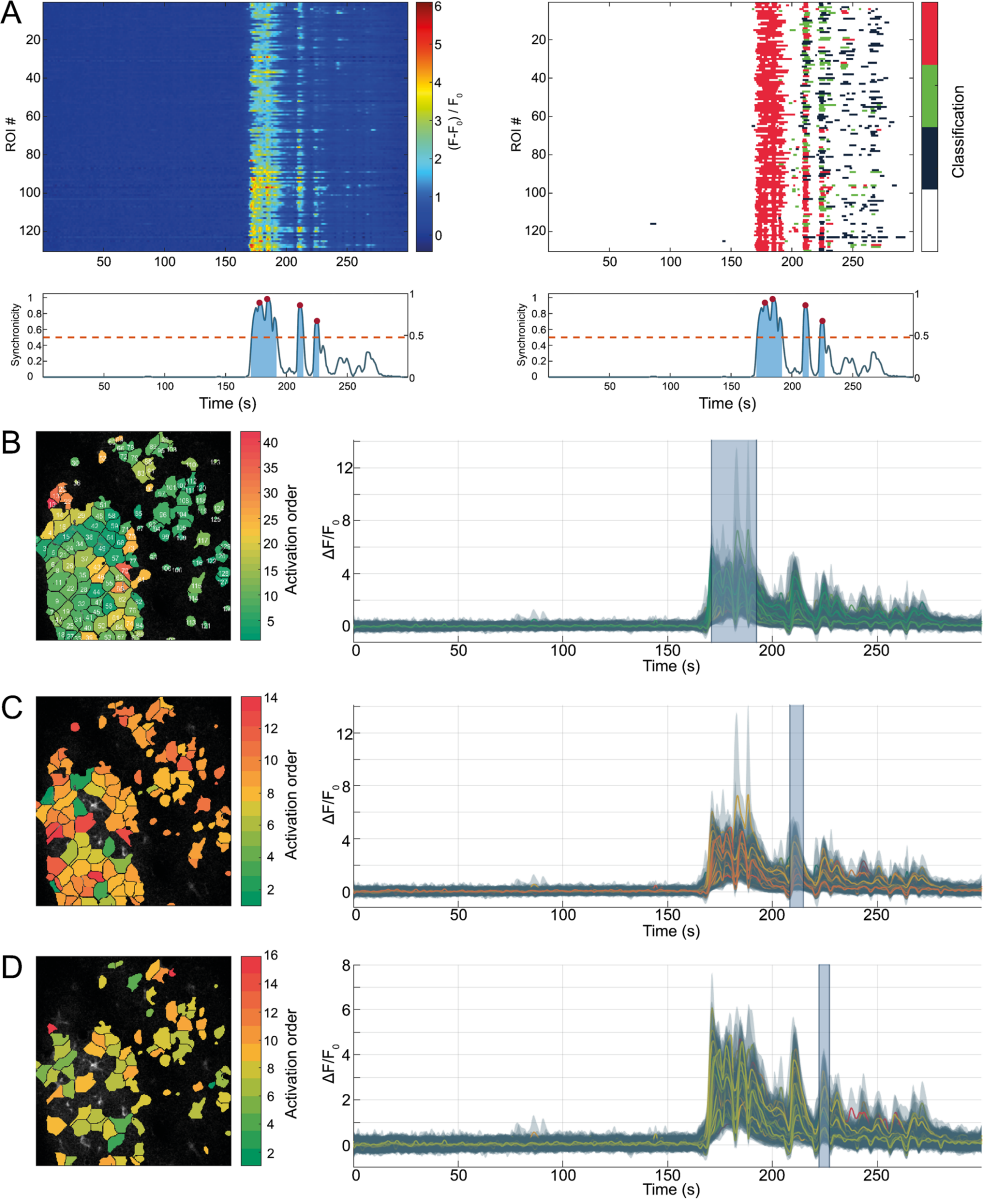
Synchronous events are diverse. Three separate but consecutive synchronous events in the same recording. Synchronous events are qualitatively assessable via kymographs, showing unclassified fluorescence profiles (**A**, left) as well as classified transients (**A**, right). In addition, synchronicity plots (**A**, beneath kymographs) allow to quantify the relative frequency of synchronous activity, with respect to the number of detected ROIs. Synchronous periods above threshold (red, dashed line) are highlighted (blue areas), and peaks in synchronicity are marked (red circles). Analyzing individual synchronous events can reveal activation patterns. Here, color indicates first activation of ROIs, from beginning (green) to end (red) of synchronous event. (**B**) The first synchronous event spread through all of the detected ROIs. (**C**) The second and third (**D**) synchronous event occured in successively smaller subsets of the detected ROIs. All three events exhibited different activation sequences, and ROIs had substantially different activation time points despite being spatially close to one another. Highlighted time-spans in the attached fluorescence profiles (**B**, **C**, **D**, right) indicate affected period of synchronicity

Astroglial networks can exhibit highly synchronized signaling behavior [24]. To detect and analyze temporally correlated events, a synchronicity index, ranging from 0 to 1, is computed. This index represents the relative frequency of simultaneously active ROIs per timepoint (Fig. 4A, bottom). A ROI is considered active during the period between the computed start and end time of an identified transient peak. Peaks in the synchronicity index and their respective duration are extracted. All ROIs exhibiting a transient peak in ∆*F*/*F*_0_ during the duration of a synchronous event are extracted together with their activation sequence. Activation sequences are determined based on the starting times of affected transients. This does not only allow identifying synchronously active regions, but also the internal activation pattern and spread of a synchronous event.

### Fluorescence imaging and data analysis

To test the algorithms, in vivo recorded Ca^2+^ signals of three transgenic mice expressing GCaMP3 were analyzed. The animals were imaged first during anesthesia and subsequently while being awake. Three to four FOVs per animal were recorded in the somatosensory cortex, resulting in a total of 23 image sequences. Similar studies investigating the effects of common anesthetics as well as natural sleep on astroglial Ca^2+^ signaling in the neocortex had previously been performed [7, 23, 32] and served as reference. First, Ca^2+^ events were analyzed in a generalized context, not differentiating between somatic events and events in the gliapil. In addition, an intersected analysis was performed. Cell somata were marked manually and intersected with the automatically detected ROIs, resulting in two distinct ROI sets. Next, the algorithms of MSparkles were compared to three other applications for Ca^2+^ analysis, CHIPS [5], CaSCaDe [1], and AQuA [33]. For this comparison, one FOV was randomly selected per mouse, and analyzed in both conditions. Finally, to demonstrate the applicability of PBasE, CoRoDe, and the analysis capabilities of MSparkles, also neuronal Na^+^ signals (SBFI ratios) obtained in acute hippocampal slices were also analyzed. In this case, ROIs were manually created in ImageJ and imported.

### Adaptive *F*_0_ estimation is able to preserve slow and long-lasting elevations of fluorescence levels

The PBasE *F*_0_ estimation algorithm presented here provides two methods for signal clean-up, a temporal mean filter and a Hampel filter, to adapt to different scenarios. In the presence of moderate fluctuations in basal fluorescence, the temporal mean filter and the Hampel filter both produce similar results (Fig. 1A). In the presence of slow, but strong increases of fluorescence levels (Fig. 1B), the Hampel filter typically incorporates these increases into the background, whereas the temporal mean filter is able to preserve such long lasting and slowly rising transients (Fig. 1B).

Pixels covered by the *F*_0_ mask (Fig. 1E) were excluded from the *F*_0_ estimation and set to their respective pre-processed time course. This results in $\frac{\Delta F}{F_0}=0$ for the affected pixels and thus prevents the detection of false ROIs and subsequently false transients. A side effect of this approach allows to gain a linear speedup of the *F*_0_ computation, corresponding to the percentage of excluded pixels.

### Accurate event detection using the CoRoDe algorithm

ROIs generated by the CoRoDe algorithm were compared against a matching set of 41 manually curated ground-truth ROIs as well as ROIs obtained using a watershed transform. For the CoRoDe algorithm as well as the watershed transform, the range projection *R* was thresholded at 0.6 ∆*F*/*F*_0_. Ground-truth ROIs were carefully outlined using ImageJ, such that the largest visible extent of a fluorescence event was captured. Areas of ground-truth ROIs were compared to the areas of the detected ROIs by computing mean differences as well as relative and absolute size differences (Table 1). Integrated ROI traces (Fig. 2C) were assessed by computing the mean signal-to-noise ratio (SNR) over all detected ROIs. Since 0.5 ∆*F*/*F*_0_ was chosen as the lowest boundary for transient classification, all values of a given ROI trace below 0.5 were considered noise.

**Table 1:** Validation of detected ROIs. ROIs detected using CoRoDe are more accurate, than those detected using a watershed transform with identical range threshold

	Ground truth	Watershed	*t*_*corr*_ = 0.5	*t*_*corr*_ = 0.25
Mean diff	0	51.2297	-21.3094	4.2631
Mean abs diff	0	51.5689	21.8059	13.8066
Mean relative diff	0	76.7984	29.7339	18.9355
Mean area (μm^2^)	59.3139	110.5436	38.0045	63.5769
Signal-to-noise ratio (db)	15.6	13.9	17.2	15.7

Absolute differences in ROI area with respect to the ground truth were significantly reduced for ROIs detected by CoRoDe, when compared to ROIs obtained by a watershed transform (Fig. 2B and D and Table 1). Furthermore, supposing an appropriate correlation threshold, here 0.25 ∆*F*/*F*_0_, ROIs obtained by the CoRoDe algorithm were found to resemble ground-truth ROIs more accurately, compared to regions obtained by watershed transform (Fig. 2B). ROIs obtained using a watershed transform were not only found to be oversized (Table 1 and Fig. 2A and B, bottom), but resulting transients were suppressed and in some cases missed (Fig. 2C, bottom). The quality of integrated ROI time profiles was assessed by comparing their SNR (Fig. 2E and Table 1). Time profiles of ROIs obtained using the CoRoDe algorithm showed no difference in SNR with respect to ground truth (Fig. 2E and Table 1). SNR is significantly reduced with time profiles of ROIs obtained using watershed transform (Fig. 2E and Table 1).

### Higher Ca^2+^ fluctuations in awake mice

In awake GCaMP3 reporter mice compared to anesthetized animals, increases in both number of detected ROIs as well as median ROI area were observed (Table 2 and Fig. 3A and B). ROIs detected in anesthetized animals were exclusively located in the gliapil. The minimal number of detected active regions was three times higher in awake animals (Table 2). In conjunction to a 50% increase of median ROI count during wakefulness, the median number of transients detected per ROI also doubled (Table 2 and Fig. 3C), resulting in the absolute number of detected events to almost double during wakefulness (Table 2 and Fig. 3J).

**Table 2:** Statistical analysis of extracted ROI and transient properties

	Anesthetized						Awake					
	ROI count	Area (μm^2^)	Transients per ROI	Frequency (Hz)	Amplitude	Duration	ROI count	Area (μm^2^)	Transients per ROI	Frequency (Hz)	Amplitude	Duration
Min	19	12.91	1	0.00824	0.5648	2.710	64	12.17	1.0	0.00813	0.5717	2.850
25%ile	45	16.39	1	0.01905	0.6693	3.192	114	19.37	1.0	0.01752	0.6506	3.600
Median	80	17.50	1	0.02847	0.7894	3.878	121	30.79	1.5	0.02025	0.7797	4.288
75%ile	116	18.74	1	0.04673	0.8702	4.273	129	150.9	2.0	0.04572	0.9850	5.963
Max	168	25.08	2	0.06256	0.9106	4.839	168	208.6	4.0	0.06322	1.8510	22.51

The transient frequency per ROI was only computed if a ROI contained more than one transient. Furthermore, the frequency per ROI was computed as the average frequency of transients between the first and the last occurrence of a transient and not as average of total number of transients divided by the recorded timespan. Investigating median transient frequencies per ROI, contrary to [32], showed no difference (0.0285 Hz and 0.0203 Hz) (Table 2 and Fig. 3D) in anesthetized and awake animals, respectively.

Investigation of transient kinetics (Table 2 and Fig. 3E–H) showed no prolongation of median transient duration during wakefulness compared to anesthetized animals (Fig. 3F). Although signaling activity was increased (Fig. 3A, C, G, and H and Table 1) during wakefulness and stronger fluctuations in fluorescence were detectable in awake animals (Fig. 3E and H), no change in overall median peak amplitude was found (Table 1 and Fig. 3E). For further analysis, transients were classified based on their peak amplitude (Fig. 3H) and assigned into one of three classes with the intervals [0.5, 1), [1, 1.5), and [1.5, ∞), respectively. This analysis revealed no difference in median amplitude among transients in the lower two classes obtained from anesthetized and awake animals. Conversely, a notable distinction was observed in the class of strongest amplitudes with awake animals exhibiting a higher median peak amplitude. Based on this classification, the signal composition was computed (Fig. 3J and Table 3). While in all transient classes the absolute number of detected transients increased, a shift in relative frequencies was revealed. In awake animals, the percentage of transients >1.5∆*F*/*F*_0_ nearly doubled, whereas the percentage of low amplitude transients decreased.

**Table 3:** Detailed statistical analysis of classified peak amplitudes

	Anesthetized			Awake		
	[0.5, 1.0)	[1.0, 1.5)	> 1.5	[0.5, 1.0)	[1.0, 1.5)	> 1.5
Min	0.5873	1.169	1.524	0.5975	1.158	1.653
25%ile	0.6444	1.181	1.688	0.6607	1.176	1.745
Median	0.6849	1.21	1.78	0.6958	1.213	1.993
75%ile	0.6974	1.217	1.942	0.7261	1.229	2.21
Max	0.7504	1.388	2.304	0.7905	1.319	2.752
Count	1166	308	156	1949	635	603

In awake mice, somatic activity could be observed in 75% of the recorded FOVs, whereas no somatic activity was recorded during anesthesia. To analyze somatic events separately from events in the gliapil, an additional ROI set was created by manually marking somatic regions. This set was then subtracted from the ROIs obtained using the CoRoDe algorithm, resulting in two distinct ROI sets that cover somata and the gliapil, respectively. Comparing somatic events to events occurring in the gliapil (Table 4 and Fig. 3K–M) revealed similar ranges of both, peak amplitude as well as transient durations in both regions. The obtained transient durations were also comparable between somatic regions and gliapil (Table 4 and Fig. 3K). Somatic transients exhibited a higher median peak compared to gliapil transients (Table 4 and Fig. 3). Analyzing raw transient counts revealed about 13 times more transients in the gliapil compared to somatic regions (Table 4 and Fig. 3M). Moreover, two-thirds of somatic transients exhibited a peak amplitude of 1.5 ∆*F*/*F*_0_ or greater, whereas half of the transients located in the gliapil had a peak amplitude < 1.0 ∆*F*/*F*_0_ (Fig. 3M).

**Table 4:** Distinct analysis of Ca^2+^ events occurring in somata and the gliapil

			Soma					Gliapil		
	Amplitude	Duration	[0.5, 1.0)	[1.0, 1.5)	> 1.5	Amplitude	Duration	[0.5, 1.0)	[1.0, 1.5)	> 1.5
Min	0.044	1.633	0.505	1.027	1.506	0.500	1.148	0.500	1.001	1.509
25%ile	1.388	3.570	0.549	1.061	2.333	0.733	3.627	0.563	1.084	1.763
Median	2.444	4.397	0.614	1.214	2.888	1.096	5.460	0.644	1.212	2.173
75%ile	3.450	8.825	0.767	1.396	3.647	1.755	10.530	0.782	1.345	2.826
Max	6.317	42.880	0.891	1.499	5.685	5.738	41.430	0.999	1.498	8.302
Count	167	167	29	26	112	2264	2264	1262	450	552

### Synchronous events are highly diverse

Ca^2+^ activity within the recorded FOV was considered synchronous if at least half of the detected ROIs were simultaneously active (Fig. 4A, bottom, dashed line). During anesthesia, no significant synchronous activity was detectable (Suppl. Table 10). In fact, no more than 20% of the detected ROIs were simultaneously active (Suppl. Table 10). During wakefulness, synchronous activity was detectable with synchronicity values exceeding 90% (Suppl. Table 10). Investigating the activation sequences of three consecutive synchronous events revealed three points: (I) Synchronous activity started from a few ROIs and then spread across the field of view (Fig. 4B–D). (II) It was neither possible to identify a predominant direction of propagation nor a repetitive propagation pattern (Fig. 4). In contrast, some regions directly adjacent to the origin of the synchronous event did not show any considerable fluorescence activity until the very end of the synchronous period (Fig. 4C). (III) During three consecutive synchronous events, there was a considerable overlap in the active ROIs, but they were never 100% identical. Moreover, the number of ROIs participating in a synchronous event degraded in consecutive events (Fig. 4B–D). Furthermore, the order of activation was different in consecutive synchronous events (Fig. 4B–D). These are only observations from a very limited number of datasets and require further investigation to obtain conclusive results.

### Analysis of Na^+^ signals

Recurrent network Na^+^ oscillations in CA1 pyramidal neurons, generated by disinhibition of the hippocampal network, were reliably analyzed by the algorithms of MSparkles (*N*=4 slice preparations from three different animals) (Suppl. figure 3). For this analysis, PBasE was used to remove trends in the ratiometric data by computing (*F* − *F*_0_). These trends were caused by accumulating phototoxic effects in one of the fluorescence channels. MSparkles’ signal analysis algorithm identified the onset of Na^+^ oscillations after wash-in of the saline containing 0 Mg^2+^/bicuculline and reported that the network essentially immediately gained a high level of synchronicity (close to 1) between all analyzed CA1 pyramidal neurons in the field of view (30–40 in a given preparation; Suppl. Figure 3A-C). Individual transients obtained from neuronal cell bodies were categorized into three groups, namely ˃5, ˃7.5, and ˃10% (corresponding to a change in 4.93, 7.39, and 9.85 mM Na^+^) (Suppl. Figure 3B). The corresponding heatmap illustrates that transients detected in individual neurons fell into all three groups, but that individual network events generally exhibited either larger (7.5 and 10%) or smaller (5 and 7.5%) peak amplitudes in the contributing cells, respectively (Suppl. Figure 3E). Peak amplitudes and durations of individual transients were comparable to results published previously (Suppl. Figure 3F, [18]). There was a weak positive linear correlation (*R*=0.36) between the peak amplitude and the overall duration of individual Na^+^ transients.

### Comparison with state-of-the-art Ca^2+^ analysis software

ROIs obtained by CoRoDe as well as transients analyzed by MSparkles were compared to data generated by three other Ca^2+^ analysis applications, CHIPS [5], CaSCaDe [1], and AQuA [33]. To obtain objective and reliable results, the official guidelines and tutorials of each application were used, and parameters were optimized. MSparkles and most of the tested applications are equipped to automatically remove false-positive ROIs. Therefore, only the number of false negatives (i.e., not detected events) was assessed.

Although AQuA and MSparkles are able to compute a much larger set of parameters, focus was set on transient kinetics (duration and peak amplitude), and the number of detected ROIs and peaks to maintain comparability. For simplicity, dynamic events detected by AQuA were considered ROIs.

### Detected kinetics are diverse among analysis tools

To compare the performance of different analysis tools (CHIPS, CaSCaDe, and AQuA), three FOVs recorded in animals expressing GCaMP3 were chosen randomly. All three FOVs in this comparison exhibit seemingly little Ca^2+^ activity during anesthesia. In the second dataset, a Ca^2+^ wave of a single astrocyte was recorded in the awake state. The third dataset contains a large Ca^2+^ wave across the entire FOV during wakefulness.

Comparing ROIs detected by different Ca^2+^ analysis applications revealed differences in ROI shape, size, smoothness, and location (Suppl. figure 4, Suppl. Table 9). Moreover, ROI segmentation greatly differs among applications (Suppl. figure 4). For example, ROIs generated by CHIPS appeared more rounded and blob-like, whereas ROIs generated by AQuA appeared rough and fragmented. Moreover, CHIPS appeared to generate larger ROIs, whereas CaSCaDe, AQuA, and MSparkles provided finer segmentations for comparable regions.

**Fig. 5:**
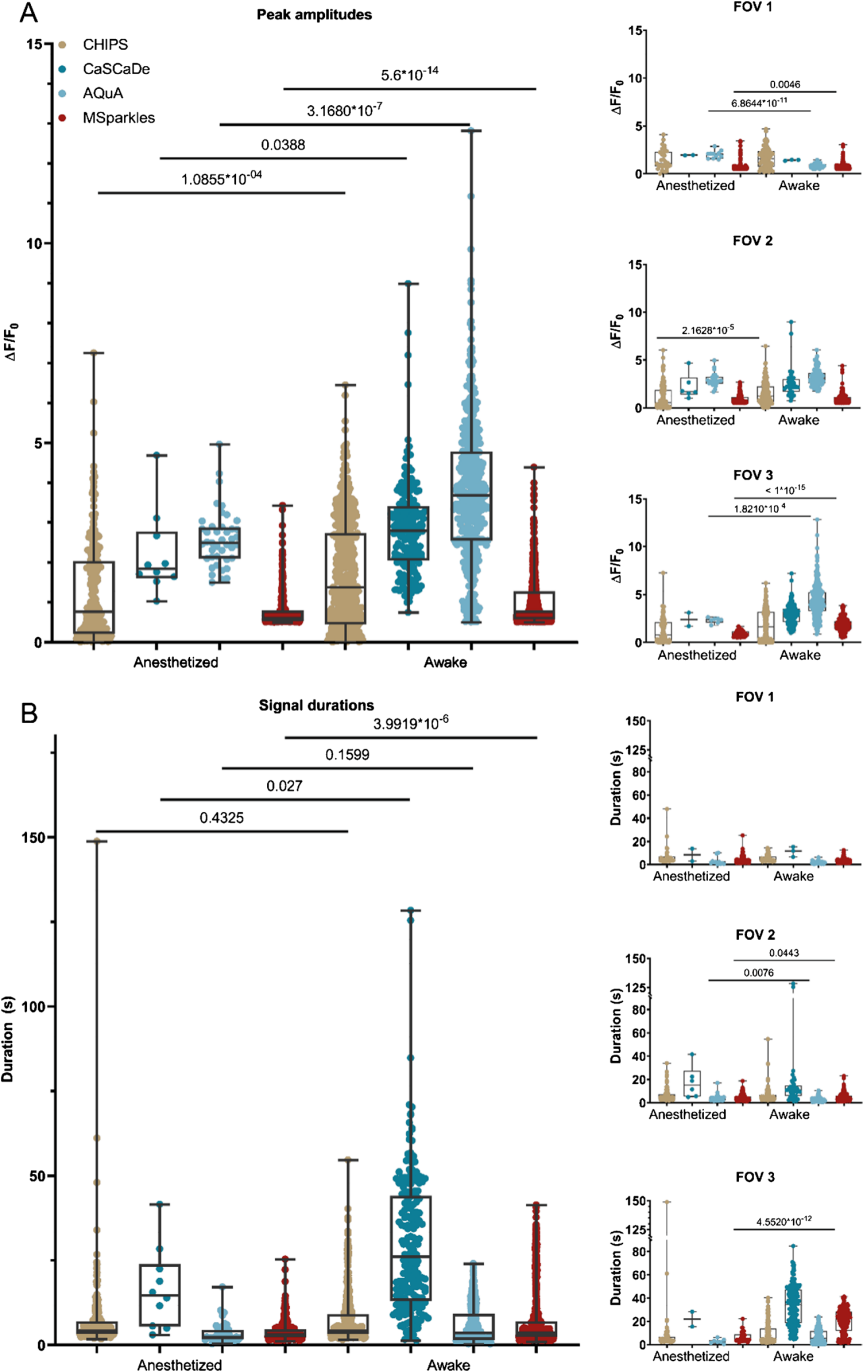
Signal kinetics obtained with different Ca^2+^ analysis tools. (**A**) Obtained peak amplitudes are heterogenous across different applications and may result in ambiguous tendencies for individual datasets between applications. (**B**) Transient durations are more consistent; however, CaSCaDe tends to measure longer durations than other applications

Overall, all applications detected a significant increase in median amplitude in awake state, compared to anesthesia (Fig. 5A). Looking at individual datasets, the determined transient peak amplitudes across the different applications appeared significantly different between anesthetized and awake states, and in some cases ambiguous (Fig. 5A and Suppl. Table 2, 3, 6). In the first dataset, CaSCaDe and AQuA found a decrease in median peak amplitude in awake state, CHIPS detected no significant difference, and MSparkles detected an increase in median peak amplitude. Similar observations were found with the second dataset. Analysis of the third dataset, exhibiting a large Ca^2+^ wave, revealed overlapping results with the tested applications. However, the magnitude of the increase in median amplitude is, again, ambiguous (Fig. 5A).

Analyzing the transient durations gave similar results (Fig. 5B and Suppl. Table 4, 5, 7). Overall, all applications detected an increased median transient duration in awake animals. However, looking at individual datasets, only AQuA and MSparkles were able of identifying significant differences between corresponding anesthetized and awake datasets (Fig. 5B). Interestingly, the transient durations reported by CaSCaDe were 2×–3× longer compared to the other applications.

### Time profiles of integrated ROIs show different signatures

**Fig. 6:**
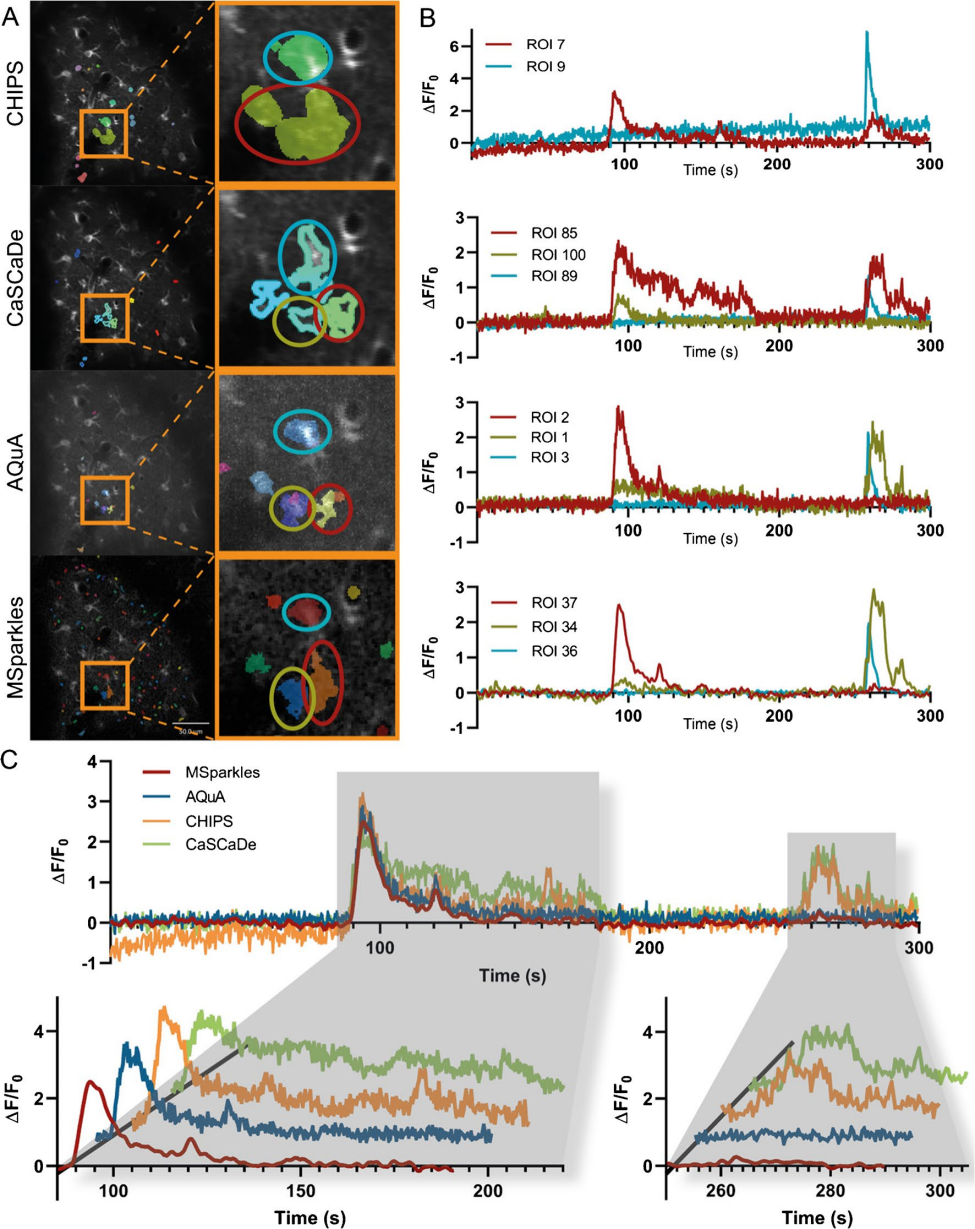
Comparison of ROI detectors. (**A**) ROIs detected by individual Ca^2+^ analysis tools. Highlighted areas contain fluorescence activity, similarly detected by all tested applications. Comparing ROIs of the magnified areas reveals segmentation differences, as well as differences in size among Ca^2+^ analysis tools. (**B**) Fluorescence profiles of magnified ROIs marked with red, yellow, and blue ellipses. Profiles obtained using CHIPS show an increase in background fluorescence over time. Especially the profiles obtained with CHIPS and CaSCaDe differ to those obtained with AQUA and MSparkles. AQuA and MSparkles performed a more accurate segmentation between the ROIs marked with red and yellow ellipses, which is reflected by the corresponding fluorescence profiles. (**C**) Direct comparison of fluorescence profiles marked by the red ellipse. Profiles are similar around the first event occurring between 90s and 180s. Profiles by CHIPS and CaSCaDe show a third peak and prolonged event, respectively. CHIPS and CaSCaDe show a second fluorescence event at around 270s. AQuA and MSparkles detected this as a separate event, located at the ROI highlighted in yellow

In order to investigate the origin of differences in the transient peaks and durations, individual time profiles of ROIs were inspected (Fig. 6A). Therefore, ROIs with a high resemblance across all applications were carefully selected. The detailed investigation of these ROIs in combination with their corresponding time profiles revealed several differences. (I) Noise levels of the integrated time profiles varied across the applications (Fig. 6A, right). (II) CHIPS resolved ROIs more coarsely and could not detrend integrated fluorescence profiles. (III) CaSCaDe appeared preferentially extracting transients with a longer duration (Fig. 6A). (IV) Integrated time profiles of AQuA and MSparkles were similar, though the profiles extracted by MSparkles appeared smoother (Fig. 6A and B).

In addition, the integrated time profiles of a single selected ROI nearly identically found by all applications were overlaid directly (Fig. 6B). All applications extracted a profile with high resemblance during the first 200 s. Especially the prominent transient peak occurring after about 100 s is mostly identical across applications.

## Discussion

We have introduced new algorithms for background correction (PBasE) and event detection (CoRoDe) and integrated them into a graphical application called MSparkles. The algorithms are designed to operate independently of microscope, fluorophores, image recording, or acquisition modality. PBasE is designed to extract information from static pixels with low-frequency fluctuations in the background fluorescence levels. CoRoDe uses the background-corrected data and detects and segments the remaining fluctuations based on user-defined parameters. Since these two algorithms are purely designed to remove and detect fluorescence fluctuations on a per-pixel level, they are not only agnostic about cell types but also about whether imaging is performed in vivo, ex vivo, or in vitro (e.g., tissue slices or organotypic cell culture). This versatility of our algorithms has been demonstrated by analyzing microglial as well as astroglial Ca^2+^ signals in the murine spinal cord in vivo and in acute slices [29]. However, there is a current limitation. Since the algorithms are developed to detect pure fluorescence fluctuations, it is currently not possible to explicitly detect and track individual cells to analyze their internal molecular signaling, but will be implemented in a future release of MSparkles. There are no explicit technical requirements on the physical resolution of the microscope (neither spatial nor temporal). These are, however, task-specific parameters and need to be adjusted based on the research object.

### *F*_0_ estimation with PBasE

Accurate estimation of fluorescence levels at basal concentrations (*F*_0_) of Ca^2+^ and other important messenger molecules is crucial to extract low amplitude transients such as microdomain events, especially in the gliapil [23]. Fluctuations in basal fluorescence levels can occur during in vivo imaging, especially in the context of long-term imaging. These variations do not necessarily occur homogeneously throughout the field of view, requiring pixel precise estimates of *F*_0_. Fitting a low-order polynomial curve to a signal before and after the occurrence of a transient shows accurate results [4]. Recently, an adaptive algorithm to automatically estimate *F*_0_ was introduced and verified by comparing it to a reference signal, recorded in a secondary fluorescence channel [23]. Approaches, based on biophysical principles [4, 23], allow to reveal the time profile of fluorescence changes [23], and make it possible to detect low amplitude events close-to-noise level. Similar to the algorithm presented in [4], the PBasE algorithm performs polynomial fitting to estimate fluorescence levels at basal Ca^2+^ concentrations. In addition, it is automated and provides two statistics-based methods to automatically exclude fluorescence transient from baseline estimation. The Hampel filter allows to closely follow slow fluctuations of given signal and can exclude relatively short peaks (Fig. 1A). Thereby, slow and long-lasting fluorescence elevations are incorporated into the estimate baseline. This may be desirable to compensate for slowly rising basal fluorescence and closely resembles the behavior presented in [23]. The temporal mean filter on the other hand is capable to preserve such slow and long-lasting elevations for later analysis. Automatic signal stabilization not only prevents high-frequency oscillations, but makes this algorithm suitable for long-term recordings.

### Automated ROI detection

In combination with PBasE, the CoRoDe algorithm was able to detect a plethora of ROIs containing low amplitude events, like microdomain events, not easily visible to a human observer and not detectable by most other Ca^2+^ analysis applications. These ROIs were predominantly located in the gliapil, where the majority of Ca^2+^ events occur [6]. The computed *F*_0_ baseline permits effectively removing slow fluctuations in background fluorescence and thus enables the extraction of subtle events, otherwise obscured by these fluctuations. In addition, the CoRoDe algorithm is capable of extracting active regions more precisely than the commonly used watershed segmentation. This in turn permits subsequent ROI integration to accurately extract transients. Using the range *R* of ∆*F*/*F*_0_ for ROI extraction has the advantage to only project actual changes in ∆*F*/*F*_0_, in contrast to using maximum or summed intensity projections which do not necessarily correspond to fluorescence events and tend to suppress weak events. However, if multiple events overlap during the recorded time period, these events might not be resolved properly and in some cases might not be detected. This is a general shortcoming of projection-based ROI detectors.

### Analysis of Ca^2+^ transients

Analyzing Ca^2+^ transients in awake and anesthetized mice showed increased ROI numbers (Fig. 3A) and ROI area (Fig. 3B) as well as transients per ROI (Fig. 3C) in awake animals. Furthermore, awake animals exhibited about twice as many Ca^2+^ transients (Fig. 3J) as well as higher peak amplitudes (Fig. 3E and G). This overall increased Ca^2+^ activity in awake animals is in line with previous studies [23, 32]. No significant decrease in the duration of Ca^2+^ transients was detected in awake animals (Fig. 3F). Instead, some animals displayed a prolonged transient duration. This might be attributable to the presence of large and long-lasting Ca^2+^ waves, which occurred exclusively in awake animals and were detected by MSparkles as individual events.

Classifying Ca^2+^ transients based on their peak amplitude allows to calculate the signal composition, i.e., the relative frequency of amplitude peaks within a defined interval. This facilitates the detection of changes in the relative incidence of the respective transient classes. Changes in signal composition can provide a more meaningful statement than just median peak amplitude. Visualizing the signal composition shows not only the overall reduction of Ca^2+^ activity, but further illustrates that this reduction happens largely on the cost of high amplitude transients, which is in line with previous work [7, 23, 32].

### Synchronous Ca^2+^ events occur during wakefulness

Synchronous Ca^2+^ event activity was defined at a threshold of 50% simultaneously active ROIs. Synchronous activity was detected exclusively during wakefulness, as observed by others [32]. Most of the synchronous Ca^2+^ activity is evoked by animal motion. Furthermore, detailed investigation of several consecutive synchronous events, these events were found to exhibit a high level of diversity. Each of the detected events showed a different activation order, and no detectable activation pattern. However, with each reoccurring synchronous event, the number of participating astrocytes decreased.

### Comparison with other software

The properties of the algorithms PBasE and CoRoDe, implemented in MSparkles, were compared to three published applications for Ca^2+^ signal analysis. All of the tested applications detected similar overall trends (Fig. 5A and B), although the mean values and value ranges differed substantially among applications. To our surprise, the individual results per FOV were quite diverse (Fig. 5A and B, Suppl. Table 2-9). Not only did the number of detected ROIs differ substantially, but also the number of extracted transients and more importantly the resulting individual trends. Investigating ROIs with high resemblance across the tested applications in combination with their corresponding time profiles provided a possible answer for this diversity. During our evaluation, CHIPS had problems in background correction. This does not only affect the scaling of signal transients when computing ∆*F*/*F*_0_, but can further generate inaccurate results when detecting and analyzing transient amplitudes (Fig. 6A, right). The ROIs subjected to further investigation integrated with CHIPS and CaSCaDe contained a secondary prominent transient peak (Fig. 6A and B). Careful analysis revealed this peak originating from a single ROI that had been segmented into two distinct ROIs by the other analysis applications. Only AQuA and MSparkles were able to resolve these ROIs properly. Despite AQuA performing dynamic event analysis and MSparkles performing a classical ROI analysis, the compared time profiles are very similar, with the difference being that profiles extracted by MSparkles appear smoother, i.e., with less noise (Fig. 6A and B).

Transient durations computed with CaSCaDe were two to three times longer than reported by any other application. Transient peaks reported by AQuA tend to exhibit a higher amplitude compared to any other analysis. However, AQuA reports fluorescence values based on local maxima, in contrast to averaged values reported by the other applications.

AQuA and MSparkles offer a graphical user interface, allowing non-programming experts to access advanced fluorescence analysis capabilities. In addition, MSparkles provides real-time visual feedback and interactive previews for data exploration and algorithm optimization. To promote a common standard within the Ca^2+^ analysis community, we provide comprehensive definitions and explanations of our terminology and computed transient properties (Supplementary Table 1, Supplementary Figure 2).

## Conclusion

To quantitatively evaluate fluorescent signal recordings of brain tissues with varying signal-to-noise ratios, vastly different fluorescence levels, recording artifacts, and diverse temporal resolutions, two novel algorithms were developed, PBasE for adaptive *F*_0_estimation and CoRoDe for the detection of fluorescence changes in stationary regions. These algorithms made it possible to identify a large number of ROIs at high spatial resolution from which close-to-noise signal (Ca^2+^ or Na^+^) transients could be determined. The analysis algorithms are embedded in a graphical user interface (MSparkles) which assists in data analysis without programming skills.

### Supplementary information

ESM 1 (DOCX 4129 kb)

## Data Availability

The software code will be available at https://gitlab.com/Gebhard/MSparkles/. The datasets generated during and/or analyzed during the current study are available from the corresponding author upon request.
