# Supplementary material for: Novel algorithms for improved detection and analysis of fluorescent signal fluctuations
Source: Pflugers Arch. 2023 Sep 13;475(11):1283–300. doi: 10.1007/s00424-023-02855-3 (PMC10567899; doi:10.1007/s00424-023-02855-3)
Supplement: Supplementary file 1 — (DOCX 4129 kb) [file 424_2023_2855_MOESM1_ESM.docx]

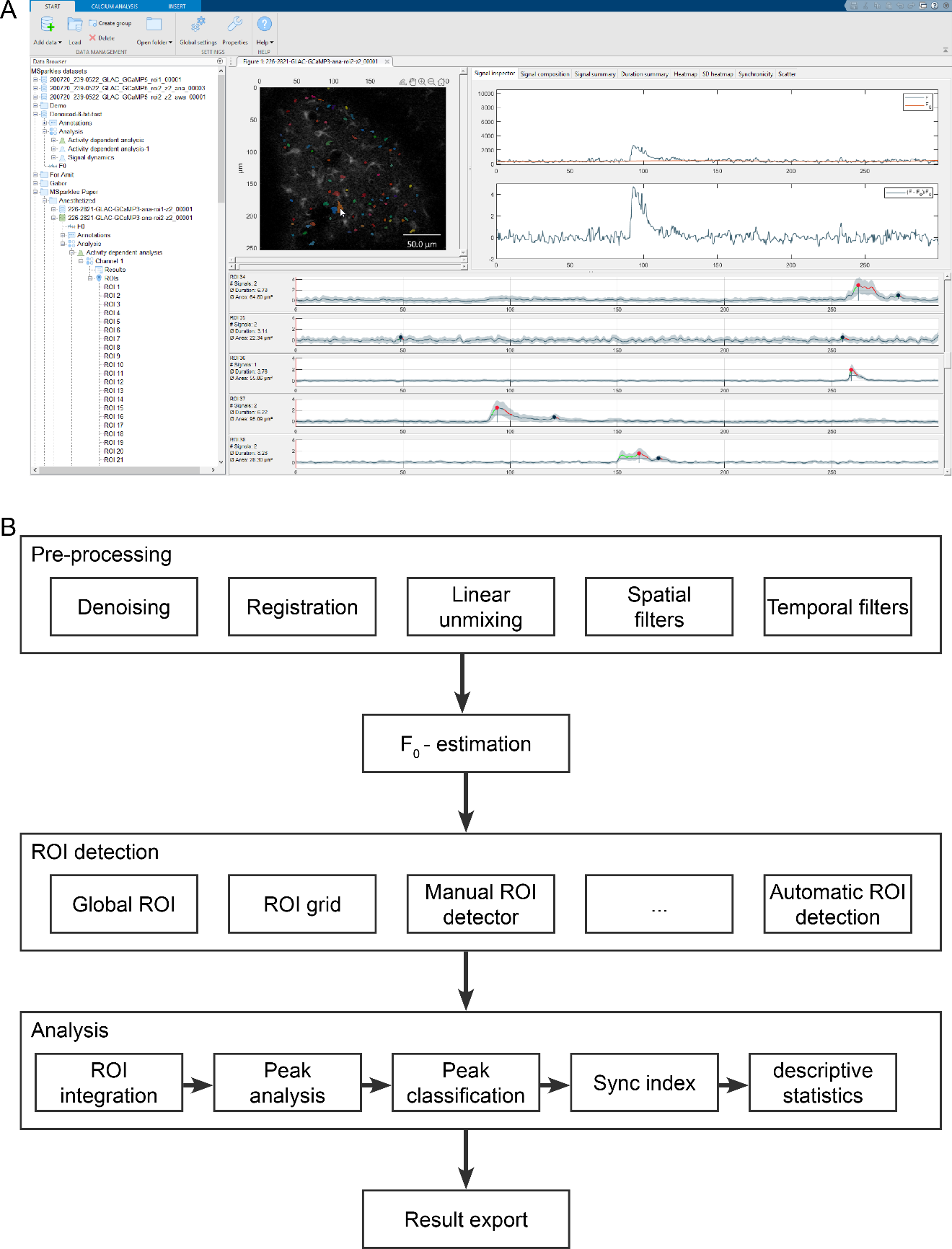


**Supplementary figure 1: User interface and processing pipeline.** A) MSparkles main user interface including hierarchical data management (left), loaded dataset with detected ROIs (center) and classified time profiles (bottom). Moving the mouse cursor across the loaded dataset, displays original and normalized time profiles (top-right). B) The Processing pipeline with individually executable and configurable pipeline stages. Pre-processing itself provides a customizable pipeline, allowing to add and remove algorithms and filters as needed. After $F_{0}$ estimation, one or more ROI detection methods can be configured. Finally, the analysis stage performs ROI integration and other downstream computations. Results, figures and graphs are automatically exported.

| *Property* | Description |
| --- | --- |
| Field of view (FOV) | Typically, a square or rectangular area, recorded by a microscope or camera system. FOVs are usually observed and recorded for a defined period of time. |
| Signal | Amplitude measurement of a physical quantity, recorded within a finite time interval at a defined sampling rate. Signals can be one- or multi-dimensional (e.g. images). |
| Amplitude | (Maximum) elevation of a signal above zero. |
| Prominence | Estimates how much a peak stands out with respect to other peaks or a non-zero reference level immediately before or after the peak. Not necessarily identical with amplitude. |
| Event | Occurrence of a temporal physiological phenomenon (e.g. local increase of intracellular [Ca^2+^], occupying a finite area or volume. |
| Wave | Special type of event with the ability to propagate through space and change its morphology. |
| Transient | Integrated, 1D representation of an event (ROI trace), identifiable as amplitude increase used for signal analysis (e.g. peak amplitude and duration measurements). |
| $\boldsymbol{F}_{\boldsymbol{org}}$ | Original, raw dataset or signal obtained from microscope. |
| $\boldsymbol{F}$ | Dataset after pre-processing. |
| $\boldsymbol{F}_{\boldsymbol{0}}$ | Fluorescence levels at basal concentrations of Ca^2+^ or other messenger molecules. |
| $\boldsymbol{\Delta F=F-}\boldsymbol{F}_{\boldsymbol{0}}$ | Background-subtracted signal. |
| $\frac{\boldsymbol{\Delta F}}{\boldsymbol{F}_{\boldsymbol{0}}}$ | Background subtracted and normalized signal. This signal is considered to be of biophysical relevance and quasi-identical to a normalized signal obtained with a secondary reference dye. |
| Fluorescence profile | Signal belonging to an individual ROI, obtained by ROI integration. The fluorescence profile of a ROI may contain zero, one or more transients. |
| Stationary event | Stable, non-moving fluorescent event, exhibiting little to no change in shape and spatial extent. This might be an inherent property of the event itself, or due to limitations of the recording technology. |
| Dynamic event | Fluorescent event, able to change its shape and location, also termed “wave”. Dynamic events can occur within a single cell or across multiple cells. |
| ROI | General term for a hand-drawn or computer-generated area of particular interest e.g. representing the extent and position of a fluorescent event. |
| Pixel trace | Entire recorded time-span of a pixel. |
| ROI trace | Signal, resulting from ROI integration. For each ROI, the average value of ${\Delta F}/{F_{0}}$ is computed for each frame. |
| Height reference | By convention typically measured as *full width at half-maximum* (FWHM). However, MSparkles also has the options to measure the duration at 25% or 10% of maximum. |
| Start / end of transient | Intersection of height reference with the rising and falling edge of a transient curve. |
| Rise time | Time difference on the rising edge between start of a transient and 90% of its peak amplitude. |
| Decay time | Time difference on the falling edge between 90% of the peak amplitude and end of transient. |
| Transient Duration | Time period between start and end of a transient. |
| Peak-to-peak time | Time interval between two consecutive transient peaks. |
| Start-to-start time | Time interval between the start of two consecutive transients. |
| Inter-transient time | Time interval between two consecutive transients. |

**Supplementary table 1: Definition of terminology**.


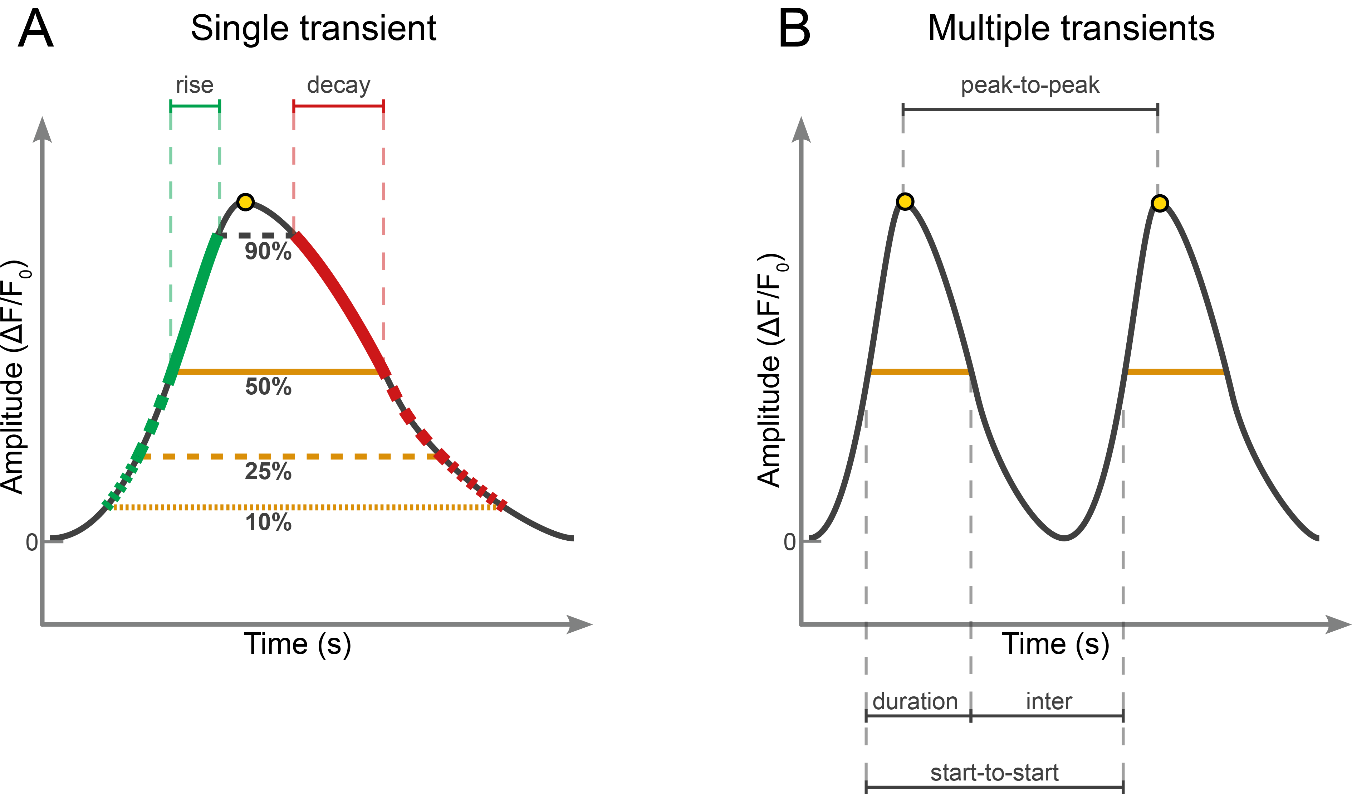


**Supplementary figure 2: Properties of Ca^2+^ transients**.

A) Properties of individual transients include peak amplitude (yellow dot), duration (orange lines) as well as rise (green) and decay times (red). Depending on the height reference, 50% (FWHM, orange line), 25%, or 10% (dashed orange lines), the duration, but also rise and decay times can be calculated more accurately. This however, requires a high signal quality. B) Consecutive transients allow to compute various signal timings, like peak-to-peak, inter signal and start-to-start. It is important to notice that these timings are influenced by the choice of the height reference.

**Supplementary figure 3: Evaluation if Na^+^ signals analyzed with MSparkles**

(A) Image of the CA1 pyramidal cell layer of a hippocampal slice (P16) stained with sodium-binding benzofuran isophthalate-AM (SBFI-AM), scale bar is 25 µm. Circles represent regions 1-5 as depicted in (B). (B) Na^+^ signals from regions 1-5 as detected during recurrent network activity. Peaks detected by the software shown by colored dots depending on threshold groups (Red >10% Green >7.5%, Blue >5%). Peak amplitude and full width at half maximum are indicated by black lines. (C) Synchronicity plot of all cells measured in the experiment shown in (A) and (B) (n=33), showing the proportion of cells with activity over time. (D) 3D plot generated by MSparkles, showing Na^+^ traces of all measured cells. (E) Threshold group heat map showing the time points at which each cell was involved in peaks with color code corresponding to that in (B). (F) Scatter plot generated by MSparkles showing the correlation between the duration and amplitude of signals.


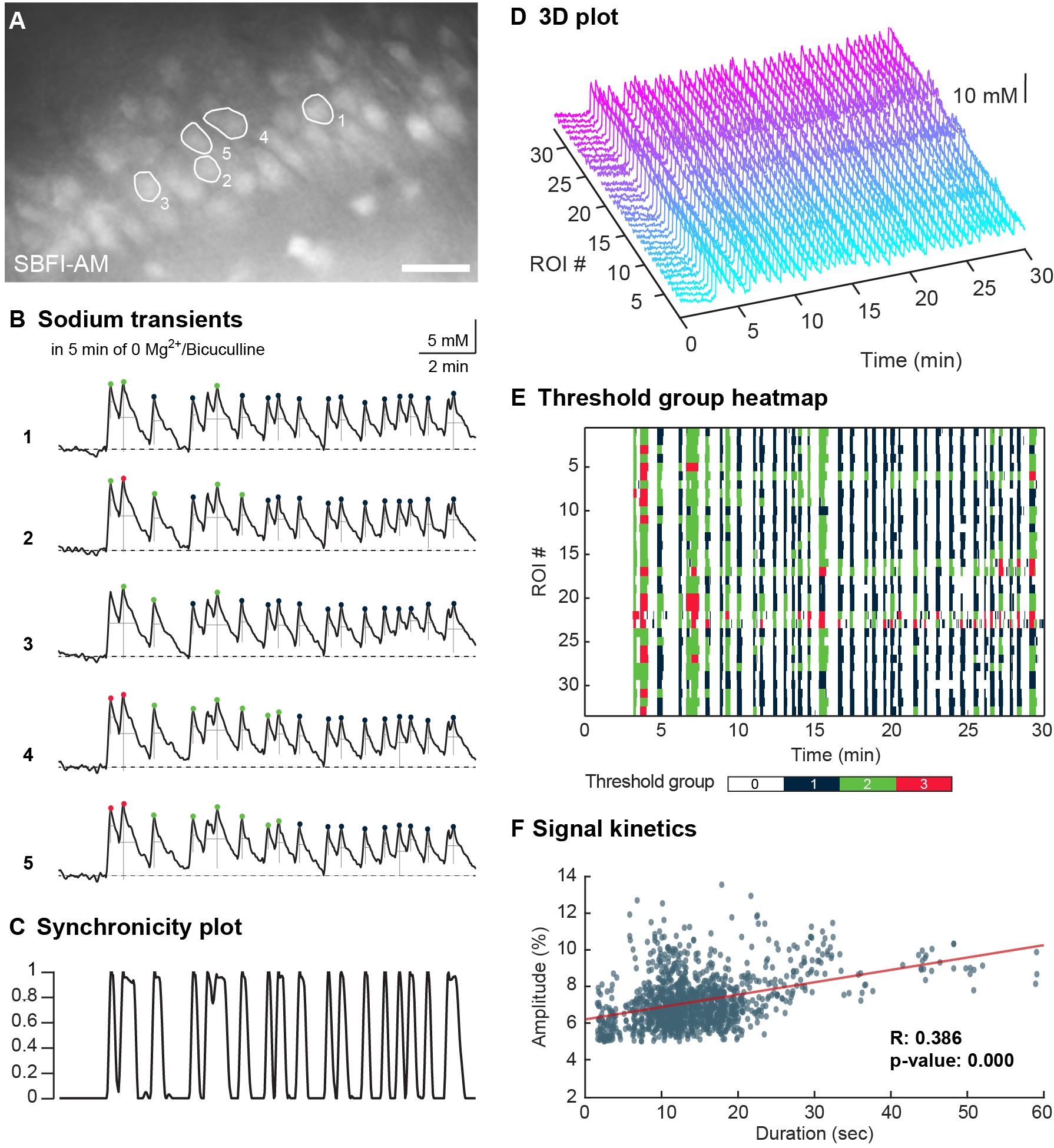


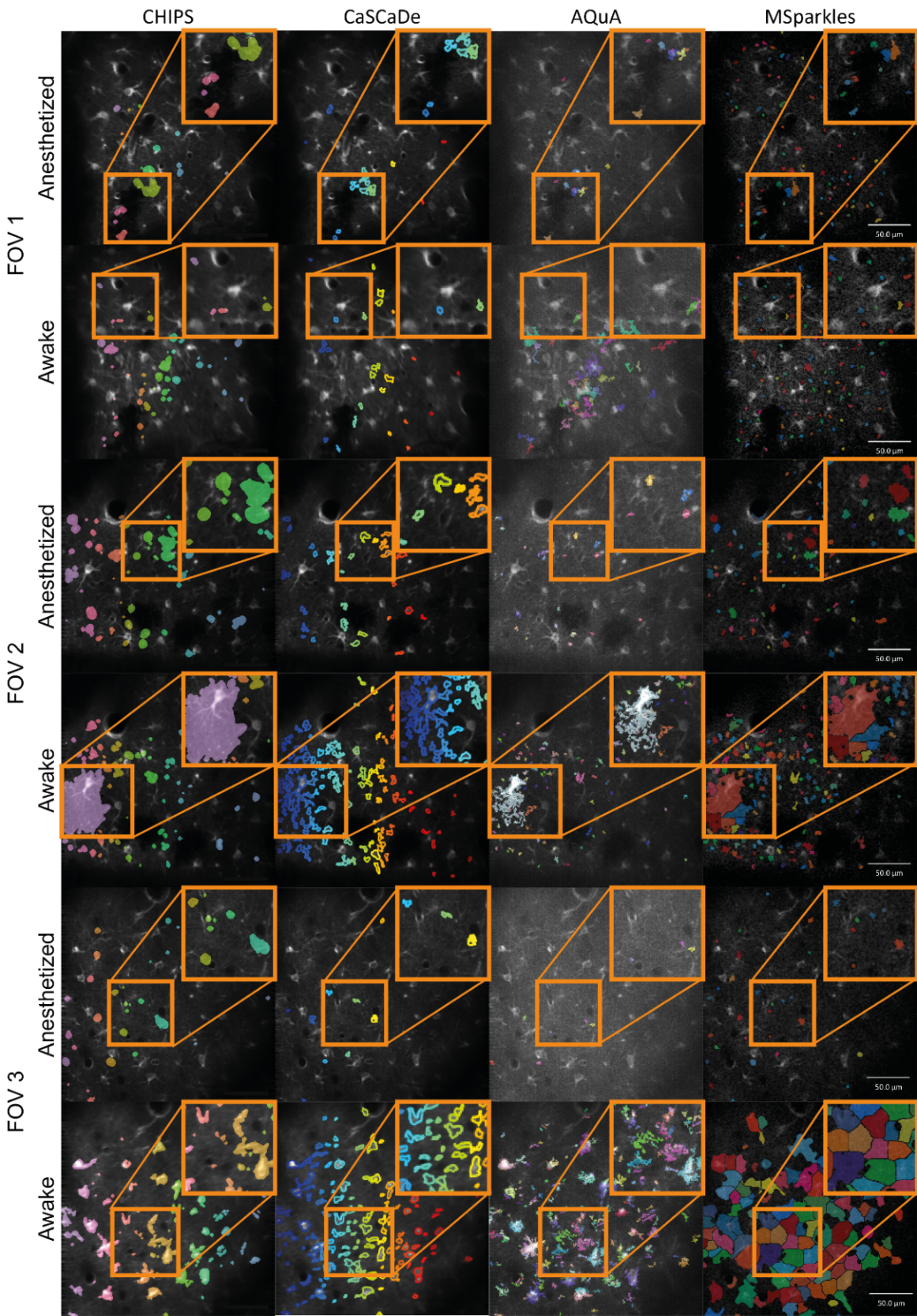


**Supplementary figure 4: Detailed comparison of ROI detectors.**

Each column shows detected ROIs obtained with a specific Ca^2+^ analysis tool. Each row is dedicated to a single FOV. Highlighted regions point out differences in detector sensitivity as well as region segmentation, potentially resulting in ambiguous measurements of ROI sizes and thus differences in ROI integration and resulting peak amplitudes. CHIPS tends to extract large and smooth regions. CaSCaDe extracts regions with a high degree of segmentation. Regions extracted by AQuA tend to be rough and contain holes. MSparkles is able to extract regions with varying smoothness, based on the temporal correlation of pixels. Due to the interplay of the PBasE and CoRoDe algorithms, MSparkles is able to detect active regions with localized and dim fluorescence events. Scale bar 50 µm.

|  | CHIPS | CaSCaDe | AQuA | MSparkles |
| --- | --- | --- | --- | --- |
| CHIPS |  | 5.683*10^-3^ | < 1*10^-15^ | 3.741*10^-5^ |
| CaSCaDe | 5.683*10^-3^ |  | >0.9999 | 3.840*10^-5^ |
| AQuA | < 1*10^-15^ | >0.9999 |  | < 1*10^-15^ |
| MSparkles | 3.741*10^-5^ | 3.84*10^-5^ | < 1*10^-15^ |  |

**Supplementary table 2: P-values between all peak amplitudes by analysis tool during anesthesia.**

P-values indicate statistically significant differences between the results obtained by different analysis applications analysing transients of anesthetized mice.

|  | CHIPS | CaSCaDe | AQuA | MSparkles |
| --- | --- | --- | --- | --- |
| CHIPS |  | < 1*10^-15^ | < 1*10^-15^ | 6.206*10^-12^ |
| CaSCaDe | < 1*10^-15^ |  | 0.2732 | < 1*10^-15^ |
| AQuA | < 1*10^-15^ | 0.2732 |  | < 1*10^-15^ |
| MSparkles | 6.206*10^-12^ | < 1*10^-15^ | < 1*10^-15^ |  |

**Supplementary table 3: P-values between all peak amplitudes during the awake state.**

P-values indicate statistically significant differences between the results obtained by different analysis applications analysing transients of awake mice.

|  | CHIPS | CaSCaDe | AQuA | MSparkles |
| --- | --- | --- | --- | --- |
| CHIPS |  | 0.0377 | 1.5643*10^-4^ | 8.2352*10^-9^ |
| CaSCaDe | 0.0377 |  | 2.5638*10^-5^ | 7.1187*10^-5^ |
| AQuA | 1.5643*10^-4^ | 2.5639*10^-5^ |  | >0.9999 |
| MSparkles | 8.2352*10^-9^ | 7.1187*10^-5^ | >0.9999 |  |

**Supplementary table 4: P-values between all signal durations during anesthesia.**

P-values indicate statistically significant differences between the results obtained by different analysis applications analysing transients of anesthetized mice.

|  | CHIPS | CaSCaDe | AQuA | MSparkles |
| --- | --- | --- | --- | --- |
| CHIPS |  | < 1*10^-15^ | 7.8676*10^-9^ | 1.0546*10^-4^ |
| CaSCaDe | < 1*10^-15^ | < 1*10^-15^ | < 1*10^-15^ | < 1*10^-15^ |
| AQuA | 7.8676*10^-9^ | < 1*10^-15^ |  | 0.1712 |
| MSparkles | 1.0546*10^-4^ | < 1*10^-15^ | 0.1712 |  |

**Supplementary table 5: P-values between all measured signal durations in awake state.**

P-values indicate statistically significant differences between the results obtained by different analysis applications analysing transients of awake mice.

| Peak amplitudes | FOV 1 | | FOV 2 | | FOV 3 | |
| --- | --- | --- | --- | --- | --- | --- |
|  | **Anesthetized** | **Awake** | **Anesthetized** | **Awake** | **Anesthetized** | **Awake** |
| CHIPS | 1.086 | 1.564 | 0.551 | 1.215 | 0.786 | 1.646 |
| CaSCaDe | 1.952 | 1.449 | 1.699 | 2.227 | 2.406 | 2.916 |
| AQuA | 2.096 | 0.849 | 2.827 | 3.079 | 2.363 | 4.157 |
| MSparkles | 0.565 | 0.595 | 0.696 | 0.845 | 0.911 | 1.851 |

**Supplementary table 6:** **Comparison of median peak amplitudes.**

Median peak amplitude per field of view, extracted by Ca^2+^ analysis applications.

| transient durations | FOV 1 | | FOV 2 | | FOV 3 | |
| --- | --- | --- | --- | --- | --- | --- |
|  | **Anesthetized** | **Awake** | **Anesthetized** | **Awake** | **Anesthetized** | **Awake** |
| CHIPS | 4.12 | 3.99 | 4.406 | 4.25 | 4.01 | 4.40 |
| CaSCaDe | 8.41 | 11.88 | 15.18 | 11.22 | 21.95 | 34.49 |
| AQuA | 1.98 | 1.32 | 2.7 | 2.10 | 2.40 | 5.55 |
| MSparkles | 2.80 | 2.85 | 3.599 | 4.02 | 4.243 | 22.51 |

**Supplementary table 7:** **Comparison of median transient durations.**

Transient duration extracted by Ca^2+^ analysis applications.

| #Rois / #fn (#sig) | FOV 1 | | FOV 2 | | FOV 3 | |
| --- | --- | --- | --- | --- | --- | --- |
|  | **Anesthetized** | **Awake** | **Anesthetized** | **Awake** | **Anesthetized** | **Awake** |
| CHIPS | 13/2 (32) | 43/7 (70) | 47/3 (139) | 54/18 (175) | 28/4 (78) | 80/23 (265) |
| CaSCaDe | 13/10 (30) | 26/23 (147) | 30/14 (50) | 127/14 (588) | 10/17 (13) | 185/11 (534) |
| AQuA | 11/11 (11) | 58/12 (58) | 24/17 (24) | 91/24 (91) | 7/20 (7) | 306/14 (306) |
| MSparkles | 85/6 (121) | 105/12 (303) | 71/8 (119) | 121/14 (266) | 29/8 (33) | 114/7 (138) |

**Supplementary table 8**: **Comparison of detected ROIs.**

Detected ROIs, false negative ROIs and signal counts. False negatives were assessed by careful manual evaluation in ImageJ for each application.

| ROI area (std. dev.) | FOV 1 | | FOV 2 | | FOV 3 | |
| --- | --- | --- | --- | --- | --- | --- |
|  | **Anesthetized** | **Awake** | **Anesthetized** | **Anesthetized** | **Awake** | **Anesthetized** |
| CHIPS | 80.20 (102.85) | 39.05 (38.08) | 75.72 (103.68) | 105.99 (442.85) | 45.35 (38.74) | 83.20 (91.35) |
| CaSCaDe | 24.42 (19.15) | 16.40 (14.02) | 24.36 (14.37) | 24.07 (15.43) | 15.73 (10.22) | 28.26 (22.86) |
| AQuA | 17.78 (21.24) | 73.97 (49.09) | 17.38 (10.53) | 24.08 (103.10) | 14.54 (4.48) | 26.89 (37.09) |
| MSparkles | 16.67 (12.35) | 14.00 (5.38) | 26.41 (23.65) | 48.36 (145.54) | 19.95 (11.07) | 218.84 (132.37) |

**Supplementary table 9:** **Mean ROI areas per FOV as detected by applications.**

Mean areas of detected ROIs with corresponding standard deviation, per FOV and condition.

|  | 2821-FOV1 | 2821-FOV2 | 2821-FOV3 | 2821-FOV3_2 | 2823-FOV1 | 2823-FOV2 | 2823-FOV3 | 2823-FOV3_2 | 4053-FOV1 | 4053-FOV2 | 4053-FOV3 | 4053-FOV4 |
| --- | --- | --- | --- | --- | --- | --- | --- | --- | --- | --- | --- | --- |
| Anesthetized | 19.79 | 9.468 | 11.59 |  | 11.2 | 8.393 | 8.333 | 8.473 | 13.56 | 15.47 | 11 | 16.29 |
| Awake | 7.748 | 15.15 | 80.39 | 71.2 | 13.18 | 9.17 | 9.691 | 98.61 | 37.52 | 36.73 | 96.78 | 35.86 |

**Supplementary table 10:** **Maximum synchronicity of Ca^2+^ signals indicated by astrocyte-specific GCaMP3-expression in genetically modified mice (in %).**

FOVs exhibiting a high synchronous activity above 50% are marked in green.
